# Right upper lobectomy in lung cancer with double aortic arch: A case report

**DOI:** 10.1111/1759-7714.13545

**Published:** 2020-06-23

**Authors:** Nobutaka Kawamoto, Riki Okita, Hidetoshi Inokawa, Masataro Hayashi, Masashi Furukawa, Masanori Okada, Kazunori Okabe

**Affiliations:** ^1^ Department of Thoracic Surgery National Hospital Organization Yamaguchi‐Ube Medical Center Ube Japan

**Keywords:** Double aortic arch, lung cancer, mediastinal lymph node dissection, recurrent laryngeal nerve

## Abstract

Double aortic arch (DAA) is a rare congenital anomaly of the heart and aorta in which a vascular ring that surrounds the trachea and esophagus is formed. In most patients, respiratory distress and dysphagia develop in childhood, and asymptomatic adult patients are rarely known. Herein, we describe a patient with lung cancer and DAA. A 66‐year‐old man who had DAA underwent video‐assisted thoracoscopic right upper lobectomy and mediastinal lymph node dissection for primary lung cancer. Lymph node dissection of the right upper mediastinum revealed that the right recurrent laryngeal nerve branched from the right vagus nerve just beneath the right aortic arch. Additionally, the right aortic arch narrowed the space surrounding the trachea, superior vena cava, and arch of the azygos vein, impeding the stapling of the truncus anterior artery and right upper lobe pulmonary vein with the video‐assisted thoracoscopic approach.

**Key points:**

**Significant findings of the study:**

- In double aortic arch, the recurrent laryngeal nerve branches from the vagus nerve just beneath the ipsilateral aortic arch.
- The right aortic arch narrows the space surrounding the trachea, superior vena cava, and arch of the azygos vein.

**What this study adds:**

- The anatomy of a double aortic arch impedes mediastinal lymph node dissection on the ventral side of the trachea. Handling autosuture devices for stapling pulmonary arteries and veins is also difficult.

## Graphical abstract

Double aortic arch (DAA) is a rare congenital anomaly of the heart and aorta in which a vascular ring that surrounds the trachea and esophagus is formed. A 66‐year‐old man who had DAA underwent video‐assisted thoracoscopic right upper lobectomy and mediastinal lymph node dissection for primary lung cancer. Lymph node dissection of the right upper mediastinum revealed that the right recurrent laryngeal nerve branched from the right vagus nerve just beneath the right aortic arch.

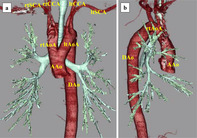

## Introduction

The incidence of anomalies in the aortic arch forming a vascular ring, such as in double aortic arch (DAA) and right aortic arch, are reported to represent 1%–2% of all congenital diseases of the heart and aorta.^1^ In most patients, DAA causes stridor, respiratory distress, and dysphagia in infancy and childhood requiring surgical treatment.^2^, ^3^, ^4^ Asymptomatic adult patients with DAA are rarely reported. Only one case report of the surgical management of a patient with lung cancer and DAA has been previously published.^5^ However, information on the surgical procedure including the course of the recurrent laryngeal nerve is insufficient. Herein, we discuss the challenges in performing right upper lobectomy and mediastinal lymph node dissection (ND2a‐1) for primary lung cancer in a patient with DAA.

## Case report

**Figure 1:**
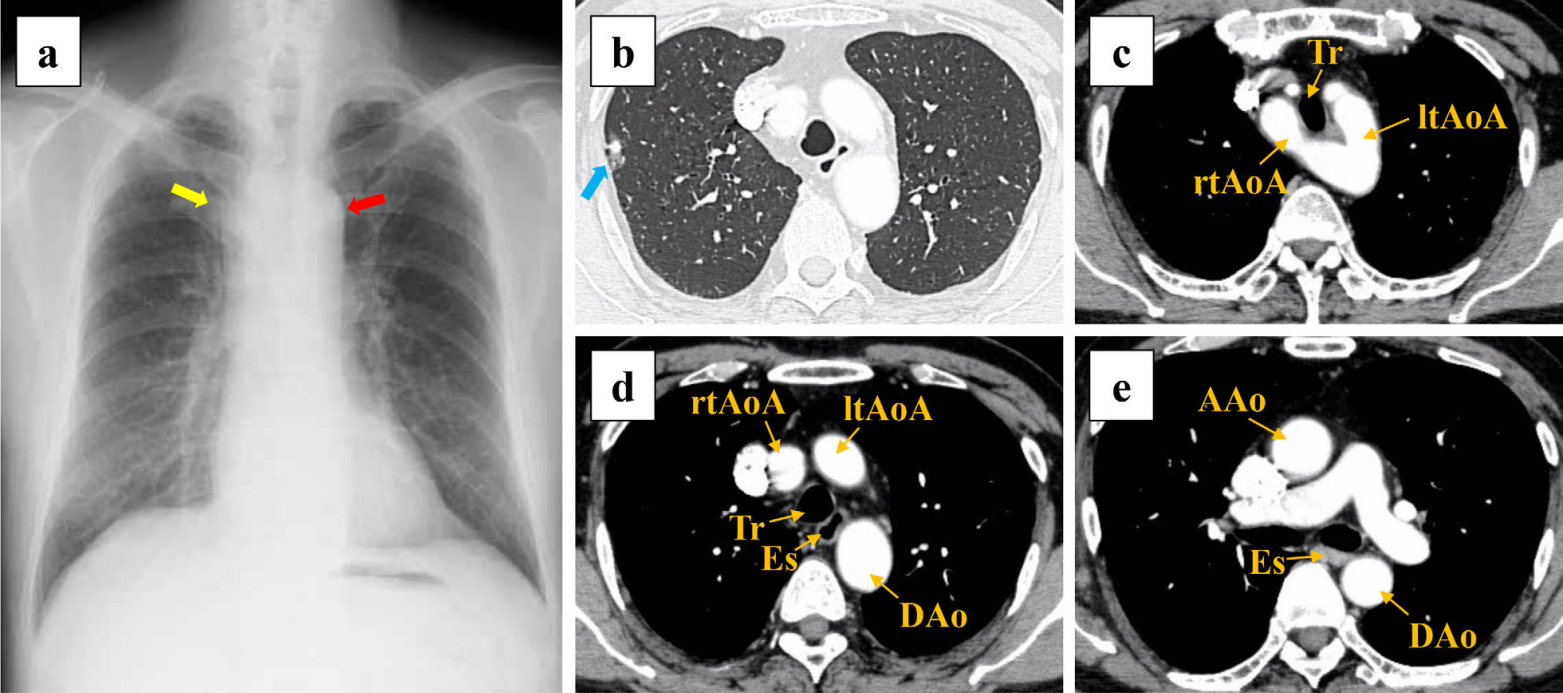
Findings of the preoperative imaging of the double aortic arch. (**a**) Chest radiography reveals bilateral aortic notches at the level of the aortic arch, suggesting aortic arch anomaly. Right aortic arch (yellow arrow) and left aortic arch (red arrow) can be seen. (**b**) Computed tomography (CT) scan shows a lung nodule in the right upper lobe (blue arrow). (**c**, **d**, **e**) Enhanced CT scan depicts a double aortic arch forming a complete vascular ring, which surrounds the trachea and esophagus. AAo, ascending aorta; rtAoA, right aortic arch; ltAoA, left aortic arch; DAo, descending aorta; Tr, trachea; Es, esophagus.

**Figure 2:**
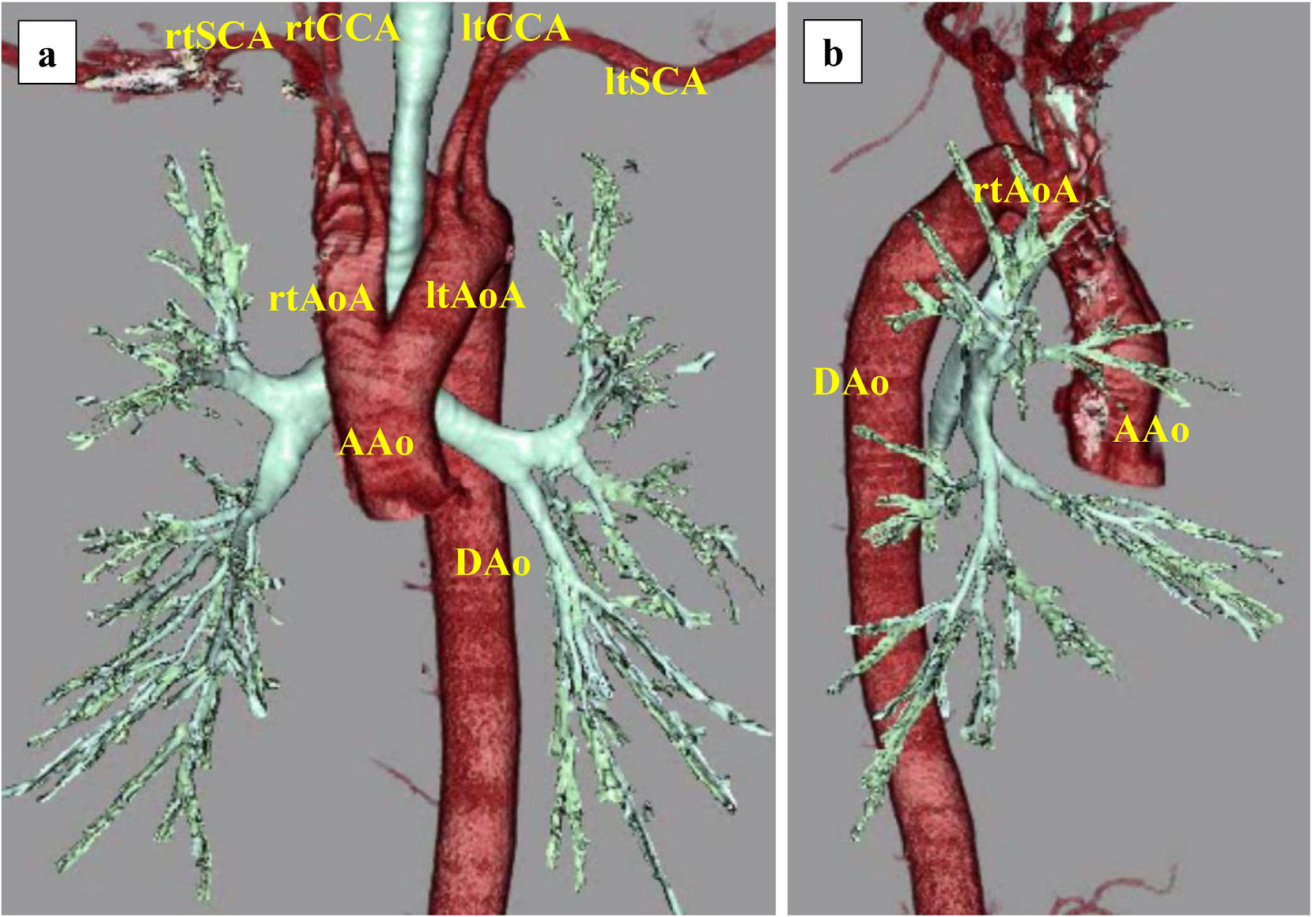
Three‐dimensional computed tomography (CT) image of the double aortic arch. (**a**, **b**) The trachea is surrounded by the left and right aortic arches. The right common carotid and right subclavian arteries originate from the right aortic arch, whereas the left common carotid and left subclavian arteries originate from the left aortic arch. AAo, ascending aorta; rtAoA, right aortic arch; ltAoA, left aortic arch; DAo, descending aorta; rtSCA, right subclavian artery; rtCCA, right common carotid artery; ltSCA, left subclavian artery; ltCCA, left common carotid artery.

A 66‐year‐old man underwent computed tomography (CT) at medical examination, which revealed a pulmonary nodule. Spirometry and the tumor marker carcinoembryonic antigen were within normal limits. Transthoracic echocardiography revealed normal heart function and no heart malformations were noted. Chest radiography revealed bilateral aortic notches at the aortic arch level, suggestive of an aortic arch anomaly (Fig 1a). CT scan revealed a 1.9 cm partly solid ground‐glass nodule (GGN) in the right upper lobe (Fig 1b), and a DAA forming a complete vascular ring around the trachea and esophagus (Fig 1c, d, e). Stenosis of the trachea or esophagus was not considerable, and the patient was completely asymptomatic. A three‐dimensional CT scan revealed the trachea was surrounded by the left and right aortic arches (Fig 2a, b). Video‐assisted thoracoscopic surgery (VATS) was performed based on the suspicion of a right upper lobe lung cancer at clinical stage IA1.

**Figure 3:**
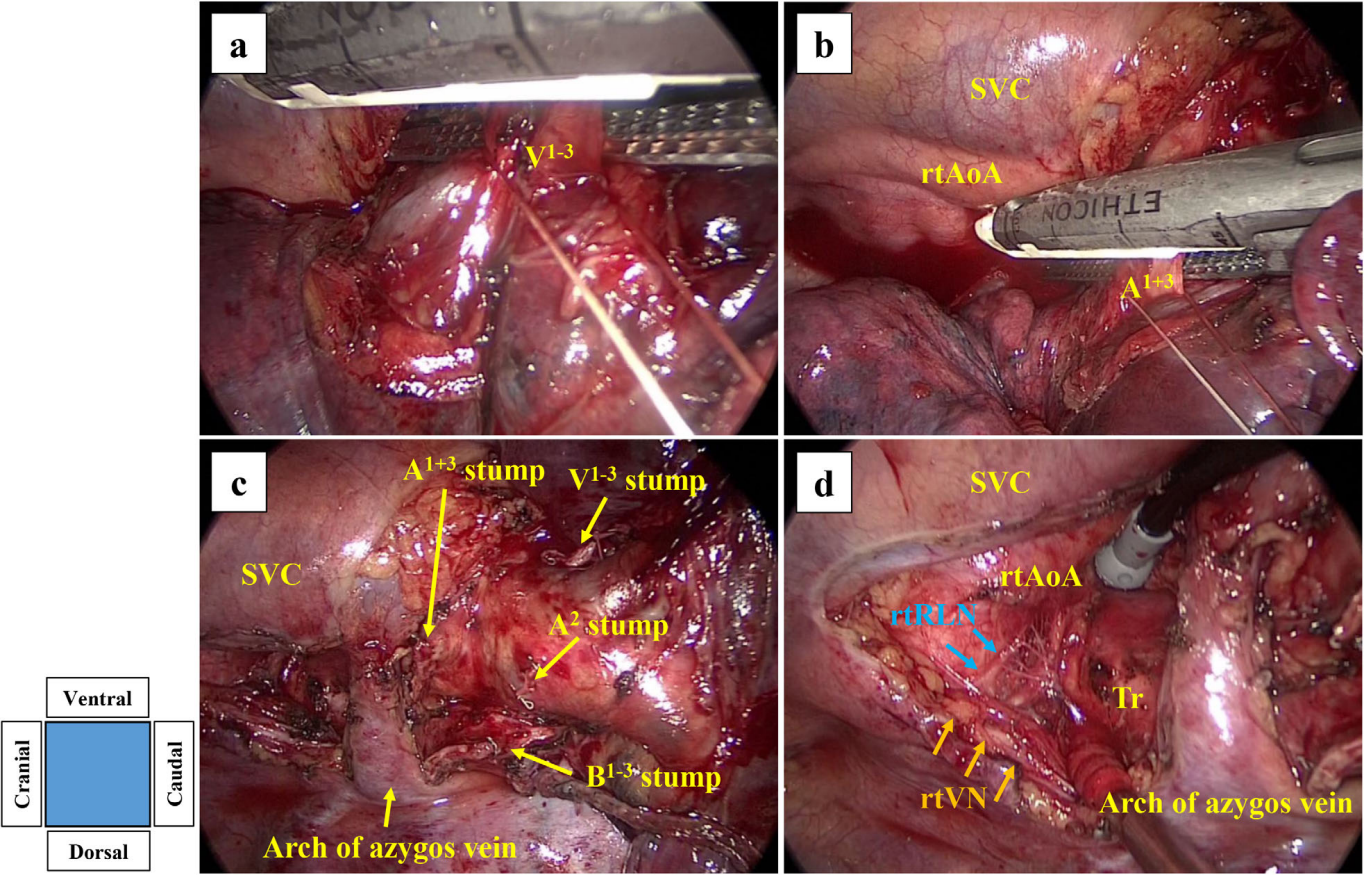
F Surgi*cal findings. (**a**) The right upper lobe pulmonary vein (V^1–3^) and (**b**) the truncus anterior artery (A^1+3^) were cut using autosuture devices. (**c**) Surgical findings after right upper lobectomy and mediastinal lymph node dissection (ND2a‐1). (**d**) The right recurrent laryngeal nerve (blue arrows) branched from the right vagus nerve (orange arrows) just beneath the right aortic arch. SVC, superior vena cava; rtAoA, right aortic arch; rtRLN, right recurrent laryngeal nerve; rtVN, right vagus nerve; Tr, trachea.*

**Figure 4:**
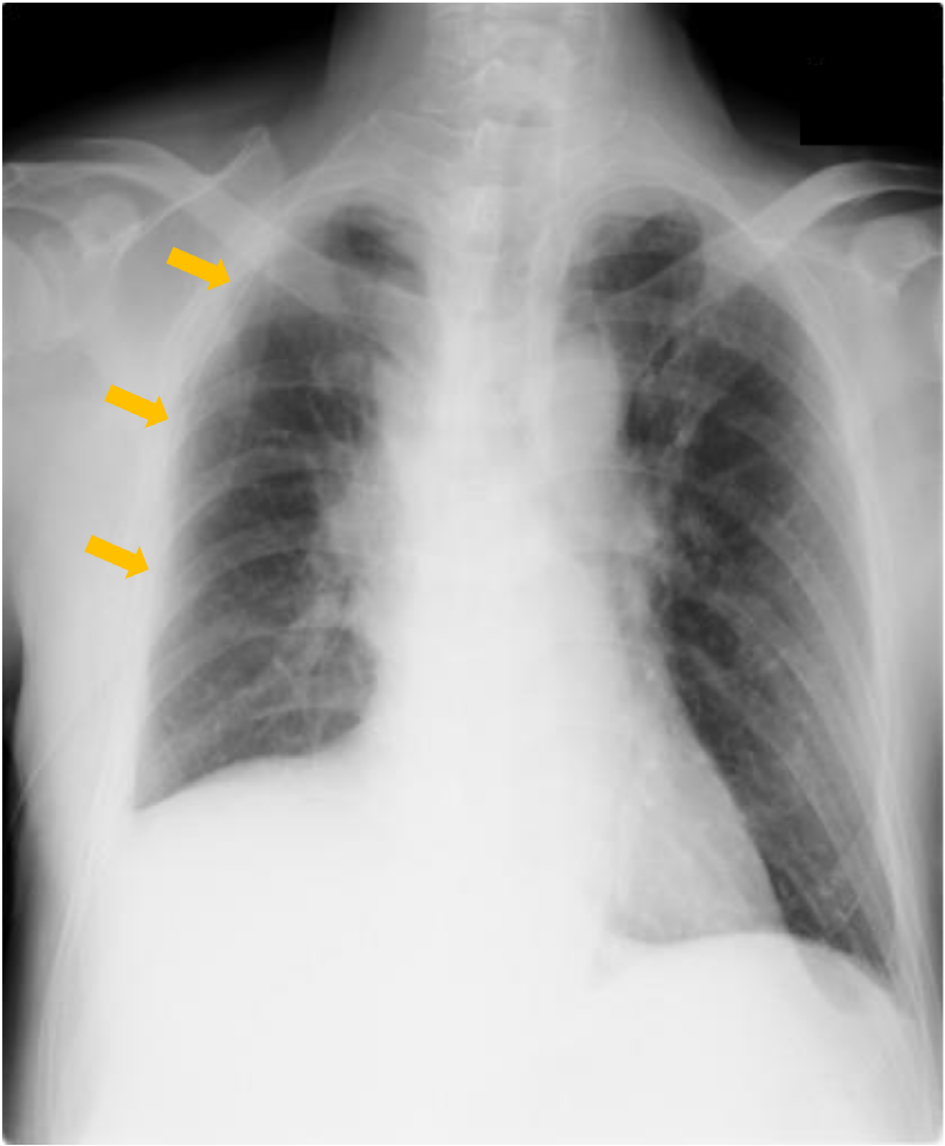
Chest radiography after right upper lobectomy on postoperative day 1. A chest drain tube (orange arrows) is placed in the right thoracic cavity. The right lung is fully inflated, and subcutaneous emphysema is not observed.

The diagnosis of the intraoperative frozen section was adenocarcinoma. The pulmonary arteries (A^1+3^, A^2^), pulmonary veins (V^1‐3^), and right upper lobe bronchus were cut using autosuture devices (Fig 3a, b), and right upper lobectomy and ND2a‐1 were performed (Fig 3c). During lymph node dissection of the upper mediastinum, the right recurrent laryngeal nerve (RLN) was revealed to branch from the right vagus nerve just beneath the right aortic arch, and No. 2R and No. 4R lymph nodes were subsequently dissected (Fig 3d). The operative time was 188 minutes and the amount of blood loss was 30 mL. A chest drain tube was placed in the thoracic cavity. No air leakage was observed postoperatively. There was approximately 200 mL/day of drainage fluid which was not bloody or chylous. Chest radiography on postoperative day (POD) 1 revealed the right lung was fully inflated (Fig 4). Therefore, the chest drain tube was removed on POD 1. The patient underwent laboratory tests and chest radiography on PODs 1, 3, and 7, and his postoperative course was uneventful. No hoarseness was observed. He was discharged on POD 10. Following pathological diagnosis, the lung adenocarcinoma was staged as IA1. After a nine month follow‐up, no recurrence of lung cancer was observed.

## Discussion

In patients with DAA, a right dorsal aortic root is present that has not regressed during the embryological process leading to the formation of a vascular ring.^6^ The ascending aorta bifurcates anterior to the trachea and esophagus, with each arch running on the left and right sides of the trachea and esophagus, respectively. These arches rejoin into a single descending aorta posterior to the trachea and esophagus, thereby encircling the two structures.^7^ DAAs are often reported to be bilaterally different; with right‐sided and left‐sided dominance in 70% and 25%, respectively, and balanced in 5%.^8^ The bifurcation from the bilateral aortic arches is symmetrical, with the common carotid arteries branching first, followed by the subclavian arteries. Patients with DAA without associated congenital heart malformations are common (83%–93%).^8^

In primary lung cancer surgery in patients with DAA, recognizing the anatomical characteristics in the upper mediastinum is essential, especially the relationship between DAA and RLN. In the previously reported case of thoracic esophageal cancer with DAA, the RLN was found to ascend along the tracheoesophageal groove in the neck, after branching from the vagus nerve just beneath the ipsilateral aortic arch.^9^, ^10^ Three‐field, D2 resection is considered standard lymphadenectomy for thoracic esophageal cancer.^11^ Paratracheal lymph nodes, namely No. 2R and No. 4R in lung cancer, are included in ND2a‐1 lymph node dissection of right upper lung cancer. However, these lymph nodes correspond to No. 106pre for esophageal cancer and are not included in three‐field D2 lymph node dissection for thoracic esophageal cancer. In lung cancer patients with DAA, lymph node dissection on the ventral side of the trachea (No. 2R and No. 4R) may not be enough due to the narrow space surrounding the trachea, right aortic arch, superior vena cava, and arch of the azygos vein (Fig 3d). This anatomical characteristic also makes it difficult to handle autosuture devices for stapling the truncus anterior artery (A^1+3^) and right upper lobe pulmonary vein (Fig 3a, b).

To the best of our knowledge, this is the first detailed report on a surgical procedure undertaken for a patient with concurrent DAA and lung cancer. Its relevance comes from the achievement of a good surgical outcome despite the potential risks due to anatomical changes.

## Disclosure

None of the authors have any potential conflicts of interest relevant to this report.

## References

[1] Kouchoukos NT , Blackstone EH , Doty DB , Hanley FL , Karp RB . Kirklin/Barratt–Boyes Cardiac Surgery, 3rd edn Churchill Livingstone, New York, NY 2003.

[2] Backer CL . Compression of the trachea by vascular rings In: Shields TW, LoCicero J, Ponn RB, Rusch VW (eds). General Thoracic Surgery, 6th edn Lippincott Williams & Wilkins, Philadelphia, PA 2004; 1082–99.

[3] Bonnard A , Auber F , Fourcade L , Marchac V , Emond S , Révillon Y . Vascular ring abnormalities: a retrospective study of 62 cases. J Pedatr Surg 2003; 38: 539–43. 10.1053/jpsu.2003.50117 12677561

[4] Backer CL , Mavroudis C , Rigsby CK , Holinger LD . Trends in vascular ring surgery. J Thorac Cardiovasc Surg 2005; 129: 1339–47. 15942575 10.1016/j.jtcvs.2004.10.044

[5] Werth SC , McLean E , Lang‐Lazdunski L . Extended right pneumonectomy in an adult with a double aortic arch: A therapeutic dilemma. Interact Cardiovasc Thorac Surg 2010; 11: 862–3. 20851875 10.1510/icvts.2010.247221

[6] Grathwohl KW , Afifi AY , Dillard TA , Olson JP , Heric BR . Vascular rings of the thoracic aorta in adults. Am Surg 1999; 65: 1077–83. 10551760

[7] Shah RK , Mora BN , Bacha E *et al* The presentation and management of vascular rings: An otolaryngology perspective. Int J Pediatr Otorhinolaryngol 2007; 71: 57–62. 17034866 10.1016/j.ijporl.2006.08.025

[8] Liang Y , Zhou Q , Chen Z . Double aortic arch with ascending aortic aneurysm and aortic valve regurgitation. Ann Thorac Surg 2014; 97: e43–5. 24484842 10.1016/j.athoracsur.2013.09.111

[9] Matono S , Fujita H , Tanaka T *et al* Esophagectomy for thoracic esophageal cancer with a double aortic arch: report of a case. Surg Today 2011; 41: 1150–5. 21773910 10.1007/s00595-010-4408-8

[10] Uemura N , Abe T , Kawai R *et al* Curative resection of esophageal cancer with a double aortic arch. Gen Thorac Cardiovasc Surg 2015; 63: 116–9. 25549887 10.1007/s11748-014-0515-6

[11] Kitagawa Y , Uno T , Oyama T *et al* Esophageal cancer practice guidelines 2017 edited by the Japan esophageal society: Part 2. Esophagus 2019; 16: 25–43. 30171414 10.1007/s10388-018-0642-8 PMC6510875

